# Supplementary material for: Venous Thromboembolism following Two Doses of COVID-19 mRNA Vaccines in the US Population, 2020–2022
Source: Vaccines (Basel). 2022 Aug 15;10(8):1317. doi: 10.3390/vaccines10081317 (PMC9414190; doi:10.3390/vaccines10081317)
Supplement: Supplementary file 1 [file vaccines-10-01317-s001.zip › vaccines-1825521-supplementary.pdf]

Supplementary to "Venous Thromboembolism following Two Doses of COVID-19 mRNA Vaccines in the US Population, 2020-2022"

Daoyuan Lai [1], Yan Dora Zhang [1,2], and Junfeng Lu [3,*]

[1] Department of Statistics and Actuarial Science, Faculty of Science, The University of Hong Kong, Hong Kong, People's Republic of China

[2] Centre for PanorOmic Sciences, Li Ka Shing Faculty of Medicine, The University of Hong Kong, Hong Kong, People's Republic of China

[3] First Department of Liver Disease, Beijing You'An Hospital, Capital Medical University, Beijing 100069, People's Republic of China

Correspondence: junfengdoc@ccmu.edu.cn

Figure S1: Flow chart of the venous thromboembolism cases showing how data from Vaccine Adverse Event Reporting System were used for Self-controlled Case Series analysis

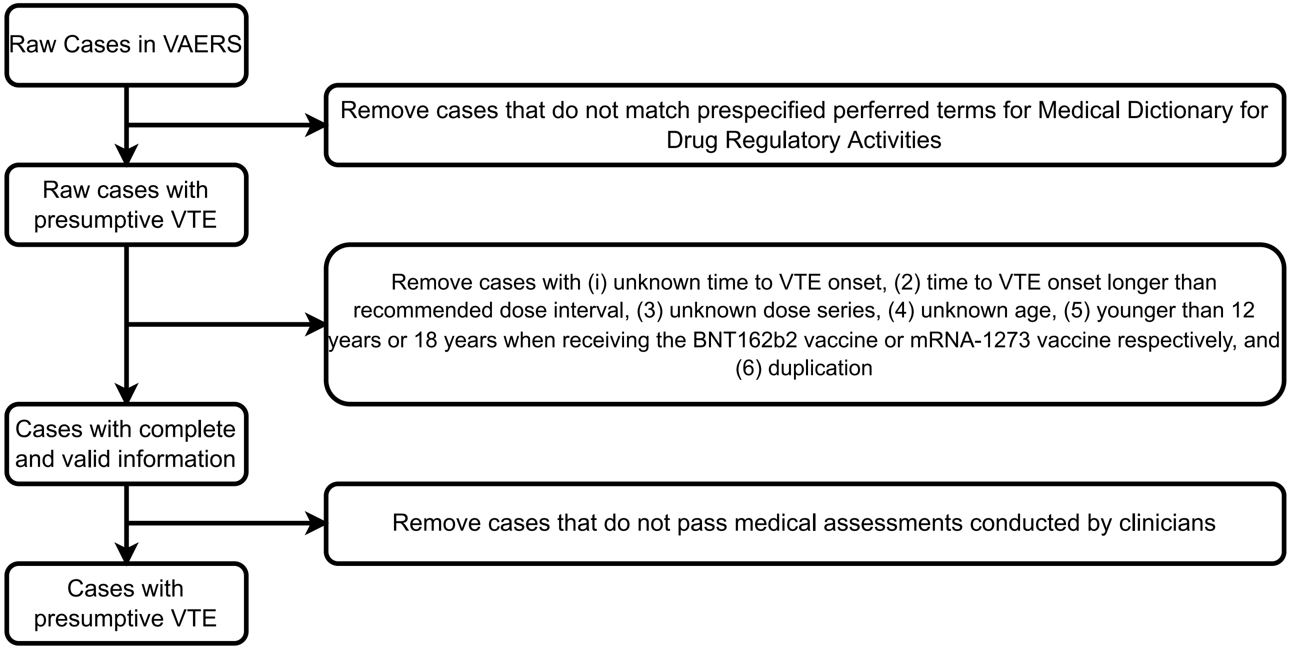

Figure S2: Visualization of the modified SCCS design using BNT162b2 vaccine as an example. Risk periods (0, 1–7, 8–14 days after COVID-19 mRNA vaccination) and the baseline periods (15-21 days after exposure) are shown. The reference period ends at day 21 as the recommended dose interval is 21 days for BNT162b2 vaccine.

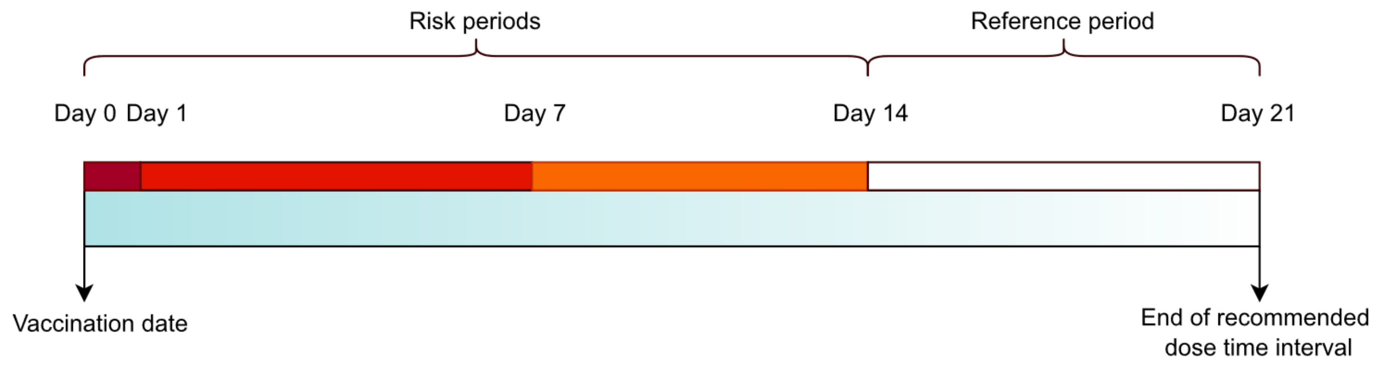

Table S1: Ratio of relative incidences and relative incidences of venous thromboembolism following the first and second dose of COVID-19 mRNA vaccination among females, stratified by age

		No. of Events[a]		RI after dose 1 (95% CI)	RI after dose 2 (95% CI)	Ratio of RIs (95% CI)[b]
		After dose 1	After dose 2			
**Female, age>=60**						
**mRNA-1273**						
	No. of days after vaccination, d					
	0[c]	11	13	0.81 (0.24, 2.66)	0.95 (0.29, 3.08)	1.18 (0.46, 3.02)
	1-7	48	51	0.69 (0.28, 1.75)	0.74 (0.3, 1.86)	1.06 (0.57, 1.98)
	8-14	29	24	0.76 (0.38, 1.5)	0.63 (0.31, 1.26)	0.83 (0.4, 1.71)
	15-28	30	28s	1 [Reference]	1 [Reference]	1 [Reference]
**BNT162b2**						
	No. of days after vaccination, d					
	0[c]	10	16	0.59 (0.12, 2.85)	0.52 (0.12, 2.28)	0.89 (0.29, 2.68)
	1-7	47	44	0.67 (0.19, 2.35)	0.35 (0.11, 1.13)	0.52 (0.22, 1.25)
	8-14	24	33	0.9 (0.37, 2.3)	0.69 (0.32, 1.52)	0.76 (0.3, 1.95)
	15-21	8	15	1 [Reference]	1 [Reference]	1 [Reference]
**Female, age<60**						
**mRNA-1273**						
	No. of days after vaccination, d					
	0[c]	18	11	1.39 (0.44, 4.47)	1.75 (0.49, 6.22)	1.26 (0.49, 3.25)
	1-7	62	59	0.93 (0.37, 2.36)	1.82 (0.68, 4.95)	1.96 (0.99, 3.87)
	8-14	32	29	0.83 (0.42, 1.64)	1.55 (0.73, 3.39)	1.87 (0.87, 4.02)
	15-28	33	14	1 [Reference]	1 [Reference]	1 [Reference]

**BNT162b2**					
No. of days after vaccination, d					
0[c]	21	23	0.70 (0.22, 2.22)	1.32 (0.4, 4.41)	1.9 (0.8, 4.52)
1-7	87	95	0.64 (0.26, 1.61)	1.21 (0.46, 3.24)	1.89 (0.94, 3.81)
8-14	37	33	0.62 (0.33, 1.19)	0.96 (0.47, 2.04)	1.55 (0.7, 3.41)
15-21	22	12	1 [Reference]	1 [Reference]	1 [Reference]

Abbreviations: CI, confidence interval; RI, relative incidence

[a] Our analysis excluded (i) reports whose time to symptom onset are unknown or exceed the recommended dose interval to ensure reports were related to a single dose, (ii) reports with unknown dose series, (iii) reports with unknown age, younger than 12 years or 18 years when receiving the BNT162b2 vaccine or mRNA-1273 vaccine respectively, and (iv) duplicated reports.

[b] The ratios are estimated by dividing the relative incidences after dose 2 by the relative incidences after dose 1.

[c] Zero refers to the day of vaccination.

Table S2: Ratio of relative incidences and relative incidences of venous thromboembolism following the first and second dose of COVID-19 mRNA vaccination among males, stratified by age

		No. of Events[a]		RI after dose 1 (95% CI)	RI after dose 2 (95% CI)	Ratio of RIs (95% CI)[b]
		After dose 1	After dose 2			
**Male, age>=60**						
**mRNA-1273**						
	No. of days after vaccination, d					
	0[c]	7	4	0.6 (0.15, 2.39)	0.20 (0.04, 0.84)	0.33 (0.08, 1.31)
	1-7	55	74	1.04 (0.37, 3.00)	0.81 (0.30, 2.17)	0.78 (0.38, 1.60)
	8-14	33	21	1.41 (0.65, 3.16)	0.52 (0.24, 1.11)	0.37 (0.16, 0.85)
	15-28	15	26	1 [Reference]	1 [Reference]	1 [Reference]
**BNT162b2**						
	No. of days after vaccination, d					
	0[c]	10	12	0.63 (0.14, 2.81)	0.28 (0.06, 1.17)	0.44 (0.14, 1.37)
	1-7	62	53	0.70 (0.21, 2.34)	0.42 (0.14, 1.28)	0.60 (0.26, 1.41)
	8-14	36	16	0.57 (0.23, 1.49)	0.68 (0.32, 1.45)	1.18 (0.45, 3.11)
	15-21	16	9	1 [Reference]	1 [Reference]	1 [Reference]
**Male, age<60**						
**mRNA-1273**						
	No. of days after vaccination, d					
	0[c]	14	3	1.36 (0.38, 4.84)	0.51 (0.09, 2.48)	0.38 (0.09, 1.51)
	1-7	45	47	0.83 (0.31, 2.30)	1.53 (0.54, 4.47)	1.84 (0.90, 3.79)
	8-14	32	22	1.00 (0.49, 2.07)	1.22 (0.54, 2.79)	1.21 (0.54, 2.71)
	15-28	27	17	1 [Reference]	1 [Reference]	1 [Reference]
**BNT162b2**						

No. of days after vaccination, d					
0[c]	4	8	0.18 (0.03, 0.84)	0.42 (0.1, 1.76)	2.35 (0.60, 9.20)
1-7	61	63	0.60 (0.21, 1.75)	0.73 (0.25, 2.16)	1.22 (0.58, 2.54)
8-14	30	24	0.67 (0.32, 1.40)	0.63 (0.29, 1.38)	0.94 (0.41, 2.18)
15-21	17	17	1 [Reference]	1 [Reference]	1 [Reference]

Abbreviations: CI, confidence interval; RI, relative incidence

[a] Our analysis excluded (i) reports whose time to symptom onset are unknown or exceed the recommended dose interval to ensure reports were related to a single dose, (ii) reports with unknown dose series, (iii) reports with unknown age, younger than 12 years or 18 years when receiving the BNT162b2 vaccine or mRNA-1273 vaccine respectively, and (iv) duplicated reports.

[b] The ratios are estimated by dividing the relative incidences after dose 2 by the relative incidences after dose 1.

[c] Zero refers to the day of vaccination.

Table S3: Ratio of relative incidences and relative incidences of venous thromboembolism following the first and second dose of COVID-19 mRNA vaccination, assuming 80% of reports can meet the Brighton Collaboration Criteria ultimately[a]

		No. of Events		RI following dose 1 (95% CI)	RI following dose 2 (95% CI)	Ratio of RIs (95% CI)[b]
		After dose 1	After dose 2			
**mRNA-1273**						
	No. of days after vaccination, d					
	0[c]	45	27	1.31 (0.67, 2.58)	1.08 (0.52, 2.23)	0.82 (0.47, 1.45)
	1-7	163	188	0.91 (0.54, 1.55)	1.44 (0.84, 2.49)	1.59 (1.10, 2.29)
	8-14	108	79	1.02 (0.70, 1.50)	1.03 (0.68, 1.57)	1.01 (0.66, 1.53)
	15-28	89	66	1 [Reference]	1 [Reference]	1 [Reference]
**BNT162b2**						
	No. of days after vaccination, d					
	0[c]	36	48	0.62 (0.29, 1.34)	0.81 (0.38, 1.72)	1.31 (0.74, 2.32)
	1-7	198	225	0.75 (0.41, 1.35)	0.83 (0.46, 1.51)	1.12 (0.73, 1.70)
	8-14	82	102	0.68 (0.44, 1.04)	0.83 (0.55, 1.26)	1.22 (0.76, 1.96)
	15-21	49	46	1 [Reference]	1 [Reference]	1 [Reference]

Abbreviations: CI, confidence interval; RI, relative incidence

[a] Our analysis excluded (i) reports whose time to symptom onset are unknown or exceed the recommended dose interval to ensure reports were related to a single dose, (ii) reports with unknown dose series, (iii) reports with unknown age, younger than 12 years or 18 years when receiving the BNT162b2 vaccine or mRNA-1273 vaccine respectively, and (iv) duplicated reports.

[b] The ratios are estimated by dividing the relative incidences following dose 2 by the relative incidences following dose 1.

[c] Zero refers to the day of vaccination.

Table S4: Ratio of relative incidences and relative incidences of venous thromboembolism following the first and second dose of COVID-19 mRNA vaccination, after excluding all decreased patients[a]

	No. of Events		RI following dose 1 (95% CI)	RI following dose 2 (95% CI)	Ratio of RIs (95% CI)[b]
	After dose 1	After dose 2			
**mRNA-1273**					
No. of days after vaccination, d					
0[c]	45	27	1.31 (0.67, 2.58)	1.08 (0.52, 2.23)	0.82 (0.47, 1.45)
1-7	163	188	0.91 (0.54, 1.55)	1.44 (0.84, 2.49)	1.59 (1.10, 2.29)
8-14	108	79	1.02 (0.70, 1.50)	1.03 (0.68, 1.57)	1.01 (0.66, 1.53)
15-28	89	66	1 [Reference]	1 [Reference]	1 [Reference]
**BNT162b2**					
No. of days after vaccination, d					
0[c]	36	48	0.62 (0.29, 1.34)	0.81 (0.38, 1.72)	1.31 (0.74, 2.32)
1-7	198	225	0.75 (0.41, 1.35)	0.83 (0.46, 1.51)	1.12 (0.73, 1.70)
8-14	82	102	0.68 (0.44, 1.04)	0.83 (0.55, 1.26)	1.22 (0.76, 1.96)
15-21	49	46	1 [Reference]	1 [Reference]	1 [Reference]

Abbreviations: CI, confidence interval; RI, relative incidence

[a] Our analysis excluded (i) reports whose time to symptom onset are unknown or exceed the recommended dose interval to ensure reports were related to a single dose, (ii) reports with unknown dose series, (iii) reports with unknown age, younger than 12 years or 18 years when receiving the BNT162b2 vaccine or mRNA-1273 vaccine respectively, and (iv) duplicated reports.

[b] The ratios are estimated by dividing the relative incidences following dose 2 by the relative incidences following dose 1.

[c] Zero refers to the day of vaccination.
